# Cellular pathology in the limbic system in schizophrenia

**DOI:** 10.1007/s00406-023-01659-x

**Published:** 2023-08-05

**Authors:** Andrea Schmitt, Peter Falkai

**Affiliations:** 1 Department of Psychiatry and Psychotherapy, LMU University Hospital, LMU Munich, Munich, Germany; 2 Laboratory of Neuroscience (LIM27), Institute of Psychiatry, University of Sao Paulo, São Paulo, SP Brazil; 3 Max Planck Institute of Psychiatry, Munich, Germany

The pathophysiology of schizophrenia (SZ) is poorly understood, especially regarding the residual cognitive dysfunction, which is difficult to treat with antipsychotic medication or psychotherapy. SZ involves structural and functional alterations in the limbic system including the anterior cingulate gyrus (ACC), and hippocampal formation. A meta-analysis across major psychiatric disorders, including SZ, gives evidence for alterations of resting-state functional connectivity in brain networks underlying cognitive dysfunction, including the ACC [1]. In individuals with SZ, limbic morphologic abnormalities have been consistently shown to be associated with cognitive deficits. At the subfield level, negative correlations between hippocampal cornu ammonis (CA) CA1, CA2/3, CA4/dentate gyrus (DG) and subiculum volumes and cognitive functioning have been demonstrated in individuals with SZ, whereby verbal memory seems to be particularly affected [2]. Both episodic and working memory deficits are related to limbic abnormalities and are hallmarks of an unfavorable outcome in SZ. As mentioned above, cognitive dysfunction drives and sustains social and vocational disability in SZ but is generally very difficult to treat.

Even patients in prodromal and first-episode stages of the disease show reduced volume, particularly in the hippocampal subfields CA4/DG [3]. However, postmortem studies in humans refuted the hypothesis of a classical neurodegenerative process in SZ by showing no neuronal loss or astrocytosis but a reduction of synaptic key proteins and a loss of oligodendroglia. The reduced number of oligodendrocytes in the CA4 subregion has been found in the anterior, posterior an entire hippocampus [4] and has recently been replicated in an independent sample [5]. These findings are in line with impaired myelination and led to the hypothesis of the role of oligodendrocytes and their precursor cells in SZ. Interestingly, cognitive deficits were associated to a loss of oligodendrocytes in the hippocampus [6]. Overall, in SZ, it has been proposed that deficient maturation of oligodendrocyte precursor cells into mature oligodendrocytes contributes to abnormal frontotemporal macro- and micro-connectivity and subsequent cognitive deficits [7]. This pathophysiological mechanism has to be proven in more detail in future postmortem and animal studies. Oligodendrocyte precursor cells, which are capable of forming new myelin sheath, may be the target of pro-myelinating drugs combined with aerobic exercise training which may foster the regeneration of myelin plasticity, thereby improving cognition. In addition, from results of postmortem and in vivo studies, impaired disease outcome in SZ may result from disturbed regenerative capacities of the brain, in particular a disturbance of synaptogenesis [8]. Aerobic exercise could not only improve myelination, but also synaptogenesis and neuronal function in brain circuits underlying cognitive domains.

However, to date, no reduced neuron number has been detected in hippocampal subfields [4, 5]. In contrast to cell density studies revealing conflicting results, the finding of a lack of neuronal loss is a result from design-based stereological studies, which are including serial brain sections and are avoiding the bias of tissue shrinkage due to the fixation or staining process. Using rigorous design-based stereology, Gaus et al. [9] in this issue report a reduction of neuron number in the whole cortical grey matter and layer V of the ACC in SZ patients compared to controls. Moreover, in the same layer, they detected a lower number of von Economo neurons (VEN). This is a completely new finding and may have impact on the pathophysiology of the disorder, since VEN are a class of specialized projection neurons, which play a critical role in social cognitive functioning. There is substantial evidence for a role of the ACC in social cognition and this cognitive domain is known to be disturbed in SZ patients. It must be mentioned that VEN neurons are only present in human beings and some higher developed animals, e.g., non-human primates, elephants and whales.

In the brain, extracellular vesicles (EVs) are secreted by neurons, microglia, and oligodendrocytes, and are involved in neuronal and cellular communication. They are also known to influence neurite growth, myelination and modulate synaptic plasticity and are involved in processes of the immune system and stress response. In a comprehensive review, Oraki Kohshour et al. [10] describe the findings in neuropsychiatric disorders including SZ and the potential use of EVs as biomarkers due to their presence in the peripheral tissues and blood. EVs contain various bioactive compounds, such as proteins, lipids, mRNAs, microRNAs, metabolites, and DNA, which makes them suitable for neurobiological research. Moreover, the EVs can cross the blood–brain barrier, and multi-omics approaches and improved isolation techniques may allow the detailed investigation of these subcellular structures in the future.

Altogether, a “human model for back-translation” to test potential remedies for cognitive dysfunction and obtaining better insight into the underlying cellular and molecular mechanisms would be of utmost importance in defining new therapeutic targets. Gaining a better mechanistic understanding of regenerative processes in the hippocampus and ACC by unraveling their pathophysiological underpinnings at the molecular and neural circuit levels could provide new targets for the development of treatments with novel modes of action that can improve illness outcome. Furthermore, it would enable development of clinically feasible, personalized treatment tools to enhance health-promoting mechanisms and counteract pathogenic ones and help to avert the debilitating outcomes of the disease such as impaired cognition.
